# Freestyle Deliberate Practice Cadaveric Hand Surgery Simulation Training for Orthopedic Residents: Cohort Study

**DOI:** 10.2196/34791

**Published:** 2022-06-29

**Authors:** Hannah K James, Ross A Fawdington

**Affiliations:** 1 Clinical Trials Unit Warwick Medical School University of Warwick Coventry United Kingdom; 2 Department of Trauma and Orthopaedics Queen Elizabeth Hospital Birmingham Birmingham United Kingdom

**Keywords:** simulation, high fidelity simulation, orthopedic residency, surgical training, postgraduate education, medical education, medical student, surgeon, hand, hand surgery, surgery, orthopedic, cadaver, cadaveric simulation, cadaveric, training, cadaveric training, DP, deliberate practice

## Abstract

**Background:**

Cadaveric simulation training may be part of the solution to reduced quantity and quality of operative surgical training in the modern climate. Cadaveric simulation allows the early part of the surgical learning curve to be moved away from patients into the laboratory, and there is a growing body of evidence that it may be an effective adjunct to traditional methods for training surgical residents. It is typically resource constrained as cadaveric material and facilities are expensive. Therefore, there is a need to be sure that any given cadaveric training intervention is maximally impactful. Deliberate practice (DP) theory as applied to cadaveric simulation training might enhance the educational impact.

**Objective:**

The objectives of this study were (1) to assess the impact of a freestyle DP cadaveric hand surgery simulation training intervention on self-reported operative confidence for 3 different procedures and (2) to assess the subjective transfer validity, perceived educational value, and simulation fidelity of the training.

**Methods:**

This study used validated questionnaires to assess the training impact on a cohort of orthopedic residents. The freestyle course structure allowed the residents to prospectively define personalized learning objectives, which were then addressed through DP. The study was conducted at Keele Anatomy and Surgical Training Centre, a medical school with an integrated cadaveric training laboratory in England, United Kingdom. A total of 22 orthopedic surgery residents of postgraduate year (PGY) 5-10 from 3 regional surgical training programs participated in this study.

**Results:**

The most junior (PGY 5-6) residents had the greatest self-reported confidence gains after training for the 3 procedures (distal radius open reduction internal fixation, flexor tendon repair, ulnar shortening osteotomy), and these gains diminished with resident seniority. The confidence gains were proportional to the perceived procedural complexity, with the most complex procedure having the lowest pretraining confidence score across all experience levels, and the greatest confidence increase in posttraining. Midstage (PGY 7-8) residents reported receiving the highest level of educational benefit from the training but perceived the simulation to be less realistic, compared to either the junior or senior residents. The most senior residents (PGY 9-10) reported the greatest satisfaction with the self-directed, freestyle nature of the training. All groups reported that they were extremely likely to transfer their technical skill gains to their workplace, that they would change their current practice based on these skills, and that their patients would benefit as a result of their having undertaken the training.

**Conclusions:**

Freestyle, resident-directed cadaveric simulation provides optimum DP conditions whereby residents can target their individualized learning needs. By receiving intensive, directed feedback from faculty, they can make rapid skill gains in a short amount of time. Subjective transfer validity potential from the training was very high, and objective, quantitative evidence of this is required from future work.

## Introduction

Cadaveric training is rapidly gaining popularity as the ultimate surgical simulation [1]. Human cadavers accurately represent anatomy as seen in the operating room, which allows residents to appreciate neurovascular and soft tissue relationships and the associated hazards with unparalleled realism [2,3]. Modern fresh-freezing techniques preserve the soft tissue handling characteristics, meaning the intraoperative “haptic feedback” when operating on cadavers is highly realistic [4]. Furthermore, when the environmental and psychological fidelities are optimized by dressing the simulation as a real operating theatre, it leads to the acquisition of both technical and nontechnical skills in a complete training package [5]—residents are not just “learning surgical skills but learning to be surgeons” 6.

Cadaveric training may be part of the solution to the joint problems of reduced quantity and quality of surgical training in the United Kingdom. The European Working Time Directive has dramatically reduced the number of hours available for surgical training [6,7], and the time that is available is not being used to best effect [8]. This is because junior residents are increasingly spending their time doing administrative and other tasks that offer less training value at the expense of attending the operating theatre [9]. Such tasks might include requesting investigations, writing discharge summaries, and other nonsurgical tasks required for their professional development such as participation in audit and quality improvement work. A large 2016 study by the Royal College of Surgeons of 990 residents found that in the average 12-hour shift, 218 minutes were spent on administrative tasks compared to just 34 minutes operating [10].

These challenges of delivering training have led to concerns about the possible patient safety implications [11]. Cadaveric simulation allows the early part of the surgical learning curve to be moved away from patients and into the laboratory so that patient safety can be assured [12]. There is a growing body of evidence that cadaveric simulation is effective for training across a wide range of specialties [1].

One known problem is that cadaveric simulation is expensive to provide [2] and is necessarily restricted to specialized wet-laboratory facilities [13]. When designing a cadaveric training course, residents will be limited to 1 attempt at each procedure [14]. It is therefore essential to maximize the impact of that training opportunity to allow for the greatest educational gains in the most cost- and time-efficient way.

Deliberate practice (DP) is an educational theory–driven way to maximize the efficiency of surgical simulation training. DP theory says that attainment of expert performance results from a continued process of targeted practice of tasks with immediate feedback, which allows learners to focus on their weaknesses while also refining other aspects of their performance [15]. It is this process, rather than merely “time on the job,” that leads to expertise, and the level of proficiency that can be attained through DP is independent of innate ability [16,17].

The aim of this study was to evaluate a “freestyle” DP cadaveric training intervention for hand surgery, where residents prospectively identified their individual learning needs using SMART (specific, measurable, achievable, realistic, time-related) objectives [18]. The course was freestyle in the sense that there was no didactic, taught element and no prescribed timetable of procedures to be performed. We hypothesize that this would provide optimum conditions for DP and would maximize and expedite the learning gains from the training.

## Methods

### Ethical Considerations

This work comes under the remit of course evaluation and therefore formal ethical board approval was not deemed necessary. The surgical training center holds the appropriate licenses to host cadaveric simulation training [13].

### Recruitment

The study was designed as a prospective cohort study. Participants were recruited via an email invitation sent to all orthopedic residents (approximately 80) in 3 regional training programs in the United Kingdom. All specialist training grades from postgraduate year (PGY) 3-10 were eligible. In total, 22 participants were recruited and completed the training.

### The Cadaveric Surgical Simulation Training Course

The training course took place over 1 day at Keele Anatomy and Surgical Training Centre. Fresh frozen whole cadaveric arms were used, obtained from the local body donation program. Instruments and implants were provided by Trimed (Trimed Inc). A large C-arm and radiographer were available to participants. The attending hand and wrist surgeon faculty each supervised 2 pairs of residents. The costs were funded by Health Education West Midlands, and the course was delivered free of cost to the participants.

Participants were asked to complete prelearning from a reading list and to write and submit bespoke SMART objectives of what they planned to achieve from the course before attending.

Participants were self-paired during the cadaveric sessions, with 2 residents to 1 cadaveric arm. Equipment to perform any or all of the procedures—distal radius open reduction internal fixation (ORIF), ulnar shortening osteotomy (USO), and flexor tendon repair (FTR)—was made available. Participants decided among themselves which procedures (or parts of procedures) they would perform. Participants were asked to pay specific attention to their SMART objectives. Attending faculty were circulating closely and were on hand to provide immediate feedback on performance. Importantly, there was no demonstration or guidance provided and no prescriptive structure to the session—the wet lab time was entirely free for the participants to explore the anatomy and perform the procedure at their own pace. This was done consciously to allow for the maximum time to be devoted to DP and is different from the usual provision in cadaveric simulation, where typically a guided demonstration is followed by participants performing all parts of all procedures in a sequential rotational manner, regardless of individual learning needs.

As part of the structured feedback and to self-audit against achievement of their SMART objectives, participants were offered the opportunity for procedure-based assessments (PBAs) to be completed by the attending faculty. PBAs are a framework for residents to receive structured feedback and allow for personal reflection.

### Data Collection and Analysis

Data were collected using prepiloted questionnaires that were designed to provide a sophisticated, subjective, and principally qualitative assessment of cadaveric simulation training 20. A Likert scale of 1-10 was used, with no middle descriptive anchor to avoid response centralization. Some questions were deliberately negativized to encourage thoughtful completion.

Demographic details and assessment of pretraining procedural confidence scores were obtained at registration before the start of the course. Posttraining confidence scores and assessment of educational value, simulator fidelity, and transfer validity potential were made at the end of the course before debriefing. Data analysis was undertaken using IBM SPSS Statistics for Windows (version 26; IBM Corp).

## Results

### Overview

There were 22 participants in the study, from PGY 5-10. Of them, 19 participants were male and 3 were female. Participants were divided into 3 subgroups for analysis, which correspond to the stages of UK higher surgical training: early (PGY 5-6), mid (PGY 7-8), and late stage (PGY 9-10). Participant demographics by subgroup are shown in Table 1. A total of 6 (28%) participants were cadaveric simulation naive, and the likelihood of past exposure to cadaveric simulation did not relate to seniority level.

**Table 1:** Participant demographics.

Characteristics		Stage of training			
		Early (PGY^a^ 5-6)	Mid (PGY 7-8)	Late (PGY 9-10)	Total
**Gender, n (%)**					
	Male	13 (93)	2 (100)	4 (67)	19 (86)
	Female	1 (7)	0 (0)	2 (33)	3 (14)
	Total	14 (100)	2 (100)	6 (100)	22 (100)
**Cadaveric simulation naive, n (%)**					
	Yes	5 (36)	0 (100)	1 (17)	6 (28)
	No	9 (64)	2 (100)	5 (83)	16 (72)
	Total	14 (100)	2 (100)	6 (100)	22 (100)

^a^PGY: postgraduate year.

### Procedural Confidence

**Figure 1:**
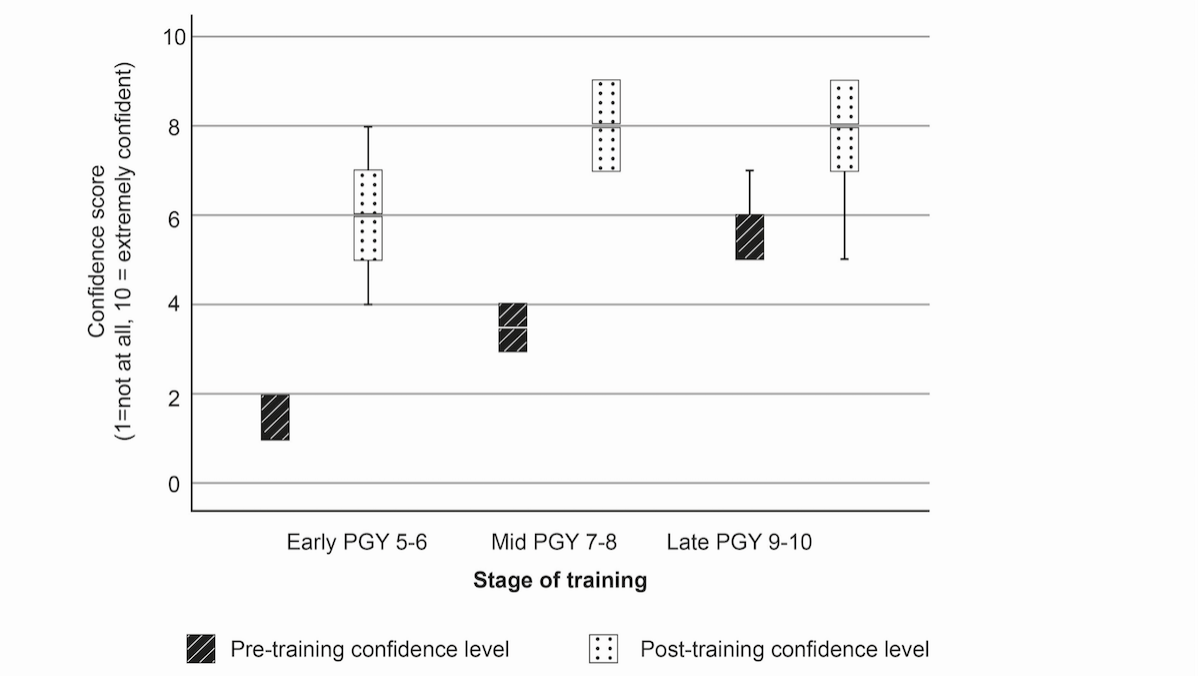
Box plot showing confidence change for ulnar shortening osteotomy. PGY: postgraduate year.

**Figure 2:**
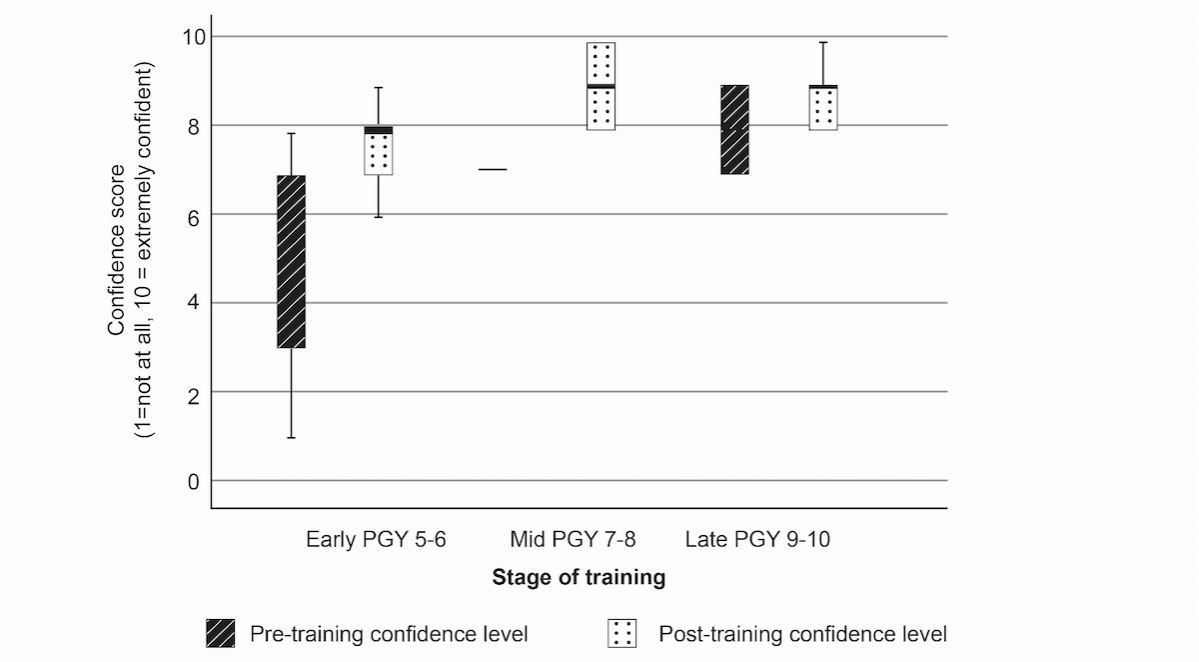
Box plot showing confidence change for distal radius open reduction internal fixation. PGY: postgraduate year.

**Figure 3:**
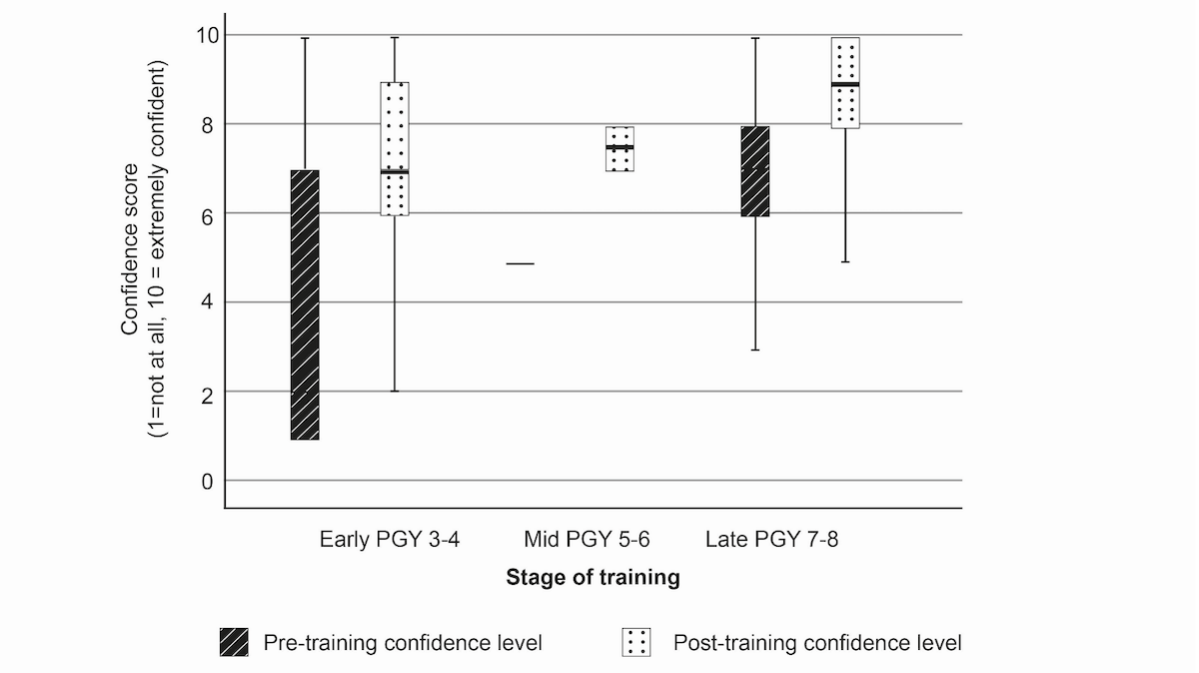
Box plot showing confidence change for flexor tendon repair. PGY: postgraduate year.

Procedural confidence increased for all procedures and within all subgroups following the DP cadaveric training. Pretraining procedural confidence was lowest for all groups for the least frequently performed procedure (USO). Mean reported confidence levels were 1.8, 3.5, and 5.2 for early-, mid-, and late-stage residents, respectively, on a Likert scale of 1-10 (where 1=not at all confident and 10=extremely confident). Posttraining confidence increased by +4.4, +4.5, and +2.5 points by subgroup (Figure 1). Pretraining confidence was highest across all subgroups for the procedure perceived to be most straightforward (distal radius ORIF), at 5.2, 7.0, and 8.0 for early-, mid-, and late-stage residents. There were confidence gains of +2.6, +2.5, and +1.7 points, respectively, after training (Figure 2). Confidence gains for FTR are shown in Figure 3.

The size of confidence gain by procedure was inversely proportional to the stage of training, with the largest gains seen in the most junior, early-stage residents (+2.3, +4.4, and +2.6 points for distal radius ORIF, USO, and FTR), moderate gains seen in the midstage residents (+2.0, +4.5, and +2.5 points), and the smallest gains seen in the most senior residents in all 3 procedures (+0.8, +2.5, and +1.7 points) (Table 2).

There were significant differences in between-group mean confidence gains for USO (*P*=.02), using 1-way ANOVA, with the most junior residents yielding the greatest gain. The between-group differences in confidence gains in distal radius ORIF and FTR were not statistically significant.

There was a significant correlation between specialist training year and mean change in confidence after training for distal radius ORIF (*P*=.01) and USO (*P*=.004) but not for FTR (Pearson test).

**Table 2:** Mean confidence gains by procedure and stage of training.

Procedure	Stage of training										
	Early (PGY^a^ 5-6)				Mid (PGY 7-8)				Late (PGY 9-10)		
	Pre	Post	Change	Pre		Post	Change	Pre		Post	Change
Distal radius ORIF^b^	5.2	7.5	+2.3	7.0		9.0	+2.0	8.0		8.8	+0.8
Ulnar shortening osteotomy	1.8	6.2	+4.4	3.5		8.0	+4.5	5.2		7.7	+2.5
Flexor tendon repair	4.1	6.7	+2.6	5.0		7.5	+2.5	6.8		8.5	+1.7

^a^PGY: postgraduate year.

^b^ORIF: open reduction internal fixation.

### Educational Value of DP Cadaveric Simulation

**Figure 4:**
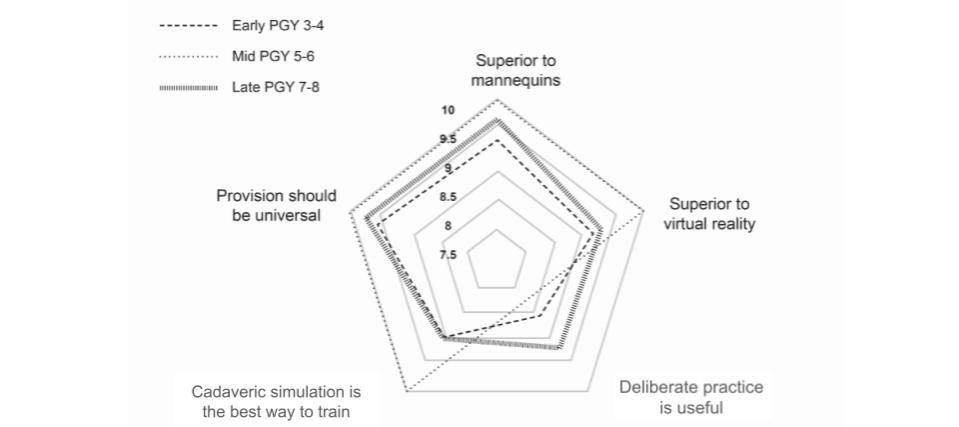
Radar plot showing educational value domain scores by training level. PGY: postgraduate year.

The perceived educational value of the training was assessed across 5 domains. All participants strongly agreed that the cadaveric training was superior to training on mannequins (mean 9.64, range 7-10 on a Likert scale of 1-10, where 1=strongly disagree and 10=strongly agree), and it was superior to training by virtual reality (mean 9.27, range 6-10). The majority of participants believed the freestyle DP nature of the course enhanced their learning, although this was not universal (mean 8.77, range 3-10). The late-stage residents were most enthusiastic about the DP design (Table 3). The participants strongly believed that cadaveric simulation training should be more widely provided to orthopedic residents (mean 9.59, range 8-10). Subgroups scores by domain are shown in Table 3 and Figure 4.

**Table 3:** Participant perception of educational value, simulator fidelity, and transfer validity of cadaveric training (scale 1-10, where 10 is considered the best score).

Participant perception		Stage of training			
		Early (PGY^a^ 5-6), mean	Mid (PGY 7-8), mean	Late (PGY 9-10), mean	Total participants, mean (range)
**Educational value**					
	Superior to mannequins	9.5	10	9.8	9.6 (7-10)
	Superior to virtual reality	9.1	10	9.3	9.3 (6-10)
	Deliberate practice is useful	8.6	8.5	9.2	8.8 (3-10)
	Cadaveric simulation is the best way to train	9	10	9	9.1 (9-10)
	Provision should be universal	9.5	10	9.7	9.6 (8-10)
**Simulator fidelity**					
	Cadavers as patients	8.7	9	8.8	8.8 (6-10)
	Surgical anatomy	9	9	9.3	9.1 (7-10)
	Hospital environment	7.2	4.4	8	7.3 (3-10)
	Multidisciplinary team	6.4	4	7.3	6.4 (1-10)
	Psychological stress	5	4.5	5.7	5.1 (1-10)
**Transfer fidelity**					
	Will take new technical skills back to workplace	9.2	10	9.8	9.5 (8-10)
	Will take new nontechnical skills back to workplace	7.1	4.5	8.2	7.1 (1-10)
	Will change current practice	8.8	9.5	9.5	9.1 (7-10)
	My future patients will benefit	9.1	9.5	9.3	9.2 (7-10)

^a^PGY: postgraduate year.

### Simulator Fidelity

**Figure 5:**
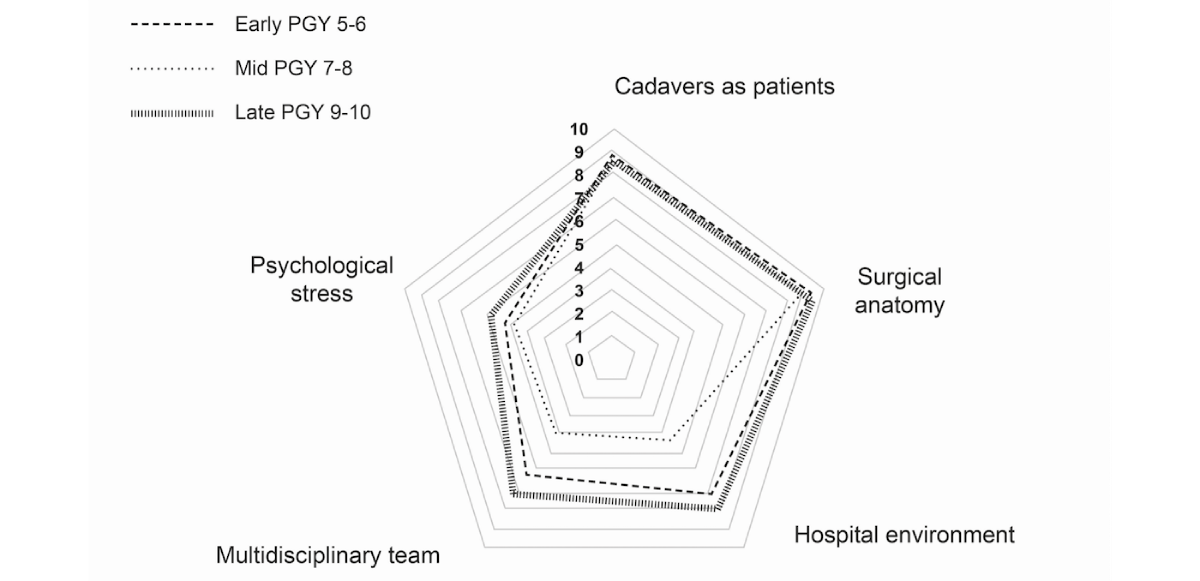
Radar plot showing simulator fidelity domain scores by training level. PGY: postgraduate year.

The fidelity of the simulation was considered across physical, environmental, and psychological domains. To assess physical fidelity, participants were asked about their perception of the realism of the cadaver as a patient and the realism of the surgical anatomy. Both were reported as being highly realistic (mean 8.77, range 6-10; and mean 9.09, range 7-10 for patient and anatomy, respectively). Environmental fidelity was assessed by asking participants about their perception of the realism of the hospital environment and multidisciplinary team. The environmental fidelity of the simulation was reported as being less than the physical fidelity but still reasonably high (mean 7.27, range 3-10; and mean 6.41, range 1-10 for hospital environment and multidisciplinary team, respectively). The psychological fidelity was assessed by asking the participants if they felt the simulation accurately recreated the emotional stress of performing real surgery. On average, the participants felt that the psychological stress of the DP cadaveric simulation was only moderately realistic, but there was a wide range of opinion on this (mean 5.14, range 1-10). The perception of psychological stress did not correlate with the stage of training. Assessment of simulator fidelity by resident stage of training is shown in Table 3 and Figure 5.

### Skill Transfer Following Training

Transfer fidelity was examined in 4 areas: transfer of technical skills and nontechnical skills to the workplace, likelihood of changing current practice following training, and belief that their future patients would benefit from participants having done the training.

All participants strongly agreed they had learned technical skills during the training that they would transfer into their surgical practice (mean 9.45, range 8-10). There was moderate agreement that nontechnical skills had been gained from the training that would transfer to the workplace, with a wide range of views (mean 7.14, range 1-10).

**Figure 6:**
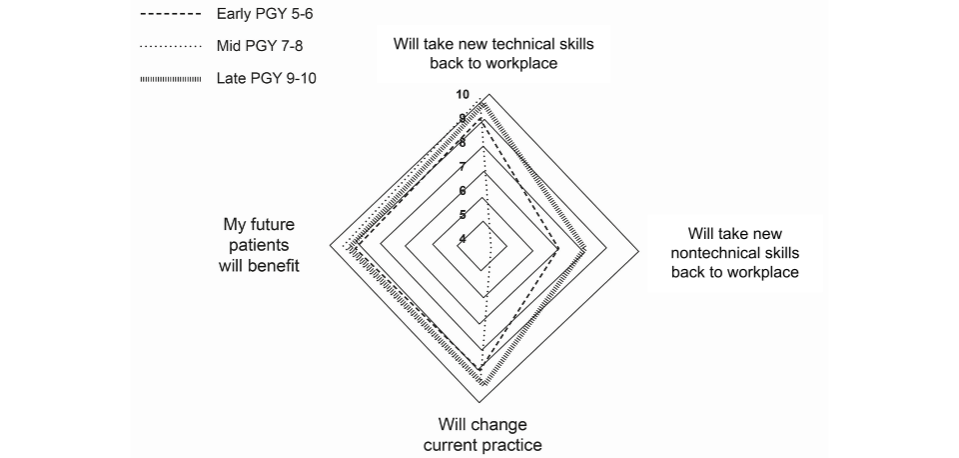
Radar plot showing transfer fidelity domain scores by training level. PGY: postgraduate year.

Participants strongly agreed they would change 1 or more aspects of their current practice based on what they had learned during the training (mean 9.05, range 7-10), and they felt that their patients would benefit from their having done the training (mean 9.18, range 7-10). The late-stage trainees overall reported the highest likelihood of skill transfer following the training (Table 3 and Figure 6).

## Discussion

### Principal Findings

The evidence base for cadaveric simulation training is growing, with an increasing number of studies showing that it can induce short-term behavioral change when measured by objective means [1]. There is still uncertainty around the timing of delivery of training and how to optimize the learning impact given the known resource limitations. We sought to assess the latter question in this study to see if a DP-style cadaveric simulation course would expedite learning and “make the most” of the single procedural attempt that is typically available in cadaveric training courses.

Procedural confidence gains following training increased with procedural complexity and were inversely proportional to experience level, with the most junior residents reporting the greatest procedural confidence gains for all 3 procedures. This is not surprising and is in line with other studies assessing the impact of cadaveric simulation on junior residents [19-29].

The most senior residents reported the greatest enthusiasm for the DP-style training. This may be because they have greater insight into their learning needs and may have greater confidence and autonomy in pursuing independent practice when compared to more junior trainees. A comparative study of standard cadaveric versus DP cadaveric training would be needed to explore this topic further.

The physical and environmental fidelities of the simulation were reported to be high by all groups, and the psychological fidelity was less so. This may be because in the cadaveric simulation laboratory, the real-world pressures of unwell patients and other clinical commitments and service pressures are absent. This lack of psychological stress with concomitant high physical and environmental fidelity has actually been shown to be a key driver of learning in cadaveric simulation [5], as participants can take time to refine their skills and learn from their mistakes in a manner that is impossible to safely replicate in the real-life operating room.

Regarding transfer fidelity, all groups reported a very high likelihood that they would take technical skills back to their workplace, but it was less so with nontechnical skills. This is not surprising as we did not design the training to develop nontechnical skills; however, evidence does show that nontechnical skills learning during cadaveric simulation may occur passively and “unnoticed” as a result of immersion in the high fidelity, “symbolically structured environment,” which exerts an “anonymous, pervasive, pedagogic action” [30]. It may, therefore, be that the participants underreported their nontechnical skills acquisition following training. There is often a considerable bias toward assessing purely technical skills following simulation training [31]; however, given that previous ethnographic studies in surgery have shown that technical skill is only thought to be around 20% of the required skill set of a competent surgeon [32,33], consideration ought to be given to the role of simulation in addressing other dimensions of competence as well.

Our study has several strengths. To our knowledge, it is the first report in the literature of the application of DP theory specifically to cadaveric simulation training. We had the full range of resident experience levels included in our study cohort, which makes subgroup assessment of impact possible. We used a sophisticated prepiloted questionnaire instrument to evaluate the course in a high level of qualitative detail across domains that are grounded in educational theory. Our study also has several weaknesses. There was no comparator group receiving “standard”’ structured cadaveric training, so any inferences we make about the likely superiority of a DP-style cadaveric training are inherently speculative. Another weakness, in common with much of the existing evidence base on cadaveric simulation, is that we used subjective, Kirkpatrick Level 1 [34] measures of impact. Objective, quantitative assessment of performance and outcome following training may provide more compelling evidence of impact, but it was not possible to do that in our study. The cohort of residents in our study were also self-selected and so may be a particularly motivated group, and hence, it is difficult to know how generalizable these results are to the orthopedic resident population as a whole. Only one-quarter of our eligible resident cohort (22 of approximately 80 residents) participated, and it is impossible to know if this represents a particular sector—perhaps those of particularly high or conversely low ability and confidence. There were only 2 residents in the midstage group, which limits the inferences that can be drawn from quantitative analysis. We chose to present 3 categories of resident seniority rather than combine groups to increase the generalizability of our results where training programs typically consider training to be in 3 phases. There was a skew toward younger and male residents in our study population, which may impact the generalizability of the results to other groups. We did not attempt to explore participants’ motivation for joining the study; it is possible that we attracted a particularly motivated cohort of participants, or the reverse may be true—individuals with low confidence in their skills may have been more likely to participate.

### Conclusions

Freestyle DP cadaveric simulation allows training efficiency and educational impact to be maximized when there are inevitable resource constraints on repeated procedural attempts. The most senior residents reported greatest enthusiasm for the DP style of training, and this may be because of a greater awareness of their own learning needs and confidence in addressing them independently. All participants reported the course to be an extremely valuable training opportunity with a very high likelihood of skill transfer to the workplace and resultant patient benefit.

## Abbreviations

DP: deliberate practice
FTR: flexor tendon repair
ORIF: open reduction internal fixation
PBA: procedure-based assessment
PGY: postgraduate year
SMART: specific, measurable, attainable, relevant, time-based
USO: ulnar shortening osteotomy
